# Publisher Correction to: Visual Computing for Industry, Biomedicine, and Art, volume 2

**DOI:** 10.1186/s42492-019-0021-x

**Published:** 2019-09-05

**Authors:** Visual Computing for Industry, Biomedicine, and Art

**Affiliations:** London, UK

**Publisher Correction to: Visual Computing for Industry, Biomedicine, and Art, volume 2**

An error occurred during the publication of a number of articles Visual Computing for Industry, Biomedicine, and Art. Several articles were published in volume 2 with a duplicate citation number.

In this correction article the old and new citation metadata are published in Table 1.

**Table 1:** Overview of incorrect and correct citation metadata

DOI	Incorrect citation number	Correct citation number
10.1186/s42492-019-0011-z	1	4
10.1186/s42492-019-0014-9	2	5

The original articles have been updated. The publisher apologizes for the inconvenience caused to our authors and readers.

